# Decreased Complications After Total Laryngectomy Using a Clinical Care Pathway

**DOI:** 10.31486/toj.20.0070

**Published:** 2021

**Authors:** Sabrina A. Brody-Camp, Sean M. Parsel, Zane A. Freeman, Edward D. McCoul, Christian Hasney, Brian A. Moore

**Affiliations:** ^1^ Department of Otolaryngology—Head and Neck Surgery, Tulane University School of Medicine, New Orleans, LA; ^2^ Department of Otorhinolaryngology, Ochsner Clinic Foundation, New Orleans, LA; ^3^ The University of Queensland Faculty of Medicine, Ochsner Clinical School, New Orleans, LA

**Keywords:** *Clinical pathways*, *laryngectomy*, *postoperative care*

## Abstract

**Background:** Complications following total laryngectomy can lead to increased hospital length of stay (LOS) and increased health care costs. Our objective was to determine the efficacy of a clinical care pathway for improving outcomes for patients following total laryngectomy.

**Methods:** This quality improvement study included all adult patients undergoing total laryngectomy—either primary or salvage—at a tertiary referral center between January 2013 and December 2018. The primary outcome was hospital LOS measured in postoperative days. The total and specific postoperative complication frequencies were evaluated, as well as 30-day readmission rates and intensive care unit (ICU) LOS.

**Results:** Sixty-three patients were included in the study: 29 (46.0%) patients before the pathway implementation and 34 (54.0%) patients after pathway implementation. Demographic characteristics between the groups were similar. The prepathway cohort had a higher rate of total complications compared to the postpathway group (relative risk=0.5; 95% CI 0.3-1.0), although the differences in individual complications were similar. The median LOS of 10 days was the same for the 2 cohorts. The median ICU LOS was 1 day greater in the postpathway cohort, but no difference was seen in rates of ICU readmission in the 2 groups. The 30-day readmission rate also was not significant between the 2 groups.

**Conclusion:** Implementation of a postoperative order set pathway for patients undergoing laryngectomy is associated with decreased overall complication rates. Use of a clinical care pathway may improve outcomes in patients undergoing total laryngectomy.

## INTRODUCTION

More than 12,000 cases of laryngeal cancer are diagnosed in the United States annually, leading to almost 3,800 deaths.^1^ Total laryngectomy is the gold standard for locally advanced laryngeal cancer and for patients who have failed primary chemoradiation therapy or conservative laryngeal surgery. Since the 1990s, the number of total laryngectomies performed nationally has declined.^2^ The decrease in total laryngectomies is in part because of a shift in the treatment paradigm toward organ preservation, either through nonsurgical treatment or minimally invasive techniques.^3-5^ In spite of this trend, more than 3,000 total laryngectomies are performed annually in the United States and are predominantly concentrated at high-volume centers.^6,7^

A patient's anatomy after a total laryngectomy is considerably altered. Breathing, communication, and swallowing functions are significantly affected by surgery. As a result, the postoperative care of laryngectomy patients can be challenging and requires a unique skill set from caretakers and health care providers. If the limitations of these patients are not recognized, devastating outcomes may result. In 2014, a sentinel event resulting in a patient death occurred at our institution. Formal review concluded that the event resulted from misunderstanding postoperative laryngectomy patient needs. In response to this incident, a multidisciplinary team convened to develop a method to prevent unintended morbidity or mortality. The solution was the implementation of a postoperative order set pathway, consisting of a set of time-released orders in the electronic medical record (EMR) that is advanced on a daily basis. The goal of the pathway is to standardize interventions and improve delivery of care. Clinical pathways such as the one used at our institution have been implemented elsewhere for a variety of conditions and have been shown to improve patient outcomes while reducing complications, costs, and length of stay (LOS).^8-12^

Beginning in January 2016, our institution implemented a laryngectomy pathway for all patients who underwent total laryngectomy. After 3 years, we sought to review how this clinical pathway affected outcomes in postoperative laryngectomy patients. Our objective was to compare patients who had had total laryngectomies at our institution prior to and after pathway implementation and to evaluate whether this intervention decreased complication rates, readmission rates, and LOS. We hypothesized that the use of the laryngectomy pathway would decrease hospital LOS and improve complication rates. To assess these outcomes, we conducted a quality improvement study of our laryngectomy population.

## METHODS

### Study Setting and Population

We conducted a quality improvement study of retrospective data for patients who underwent total laryngectomy for oncologic indications by 5 head-and-neck surgeons at a tertiary referral center in Southeast Louisiana between January 2013 and December 2018. Patients were eligible for inclusion if they had a total laryngectomy for squamous cell carcinoma involving the larynx. Inclusion criteria were based on current procedural terminology (CPT) codes corresponding to total laryngectomy without radical neck dissection (CPT 31360) and total laryngectomy with radical neck dissection (CPT 31365). Patients were excluded from the study if they were <18 years of age or underwent laryngectomy for other than an oncologic etiology.

The population was separated into 2 cohorts depending on the use of the laryngectomy pathway. The pathway was implemented on January 1, 2016, and all patients presenting for laryngectomy after this date received postoperative treatment according to the pathway. Thus, the prepathway cohort included patients receiving total laryngectomy between January 2013 and December 2015, while the postpathway cohort included patients receiving total laryngectomy between January 2016 and December 2018.

### Laryngectomy Pathway

The laryngectomy pathway order set was created to address orders commonly used with all laryngectomy patients. Once admitted after a laryngectomy, patients are automatically enrolled. The pathway is a time-dependent order set that is electronically released on subsequent postoperative days depending on patient progression and consists of standard orders on postoperative days 0 through 7 (Figure, Table 1). The patient's nurse allows release of each day's order set manually. This order set includes labs, imaging, early consultation for social work for home durable medical equipment, physical therapy and occupational therapy, and speech and language pathology. Nursing communication orders include placing a sign above the patient's bed that reads “obligate neck breather” and a wristband that reads the same. Also included is an impaired communication protocol that requires a staff member to physically present to the patient's room whenever the call button is pressed. Orders such as tube feeding formula require manual entry depending on the patient and nutrition needs.

**Figure.**
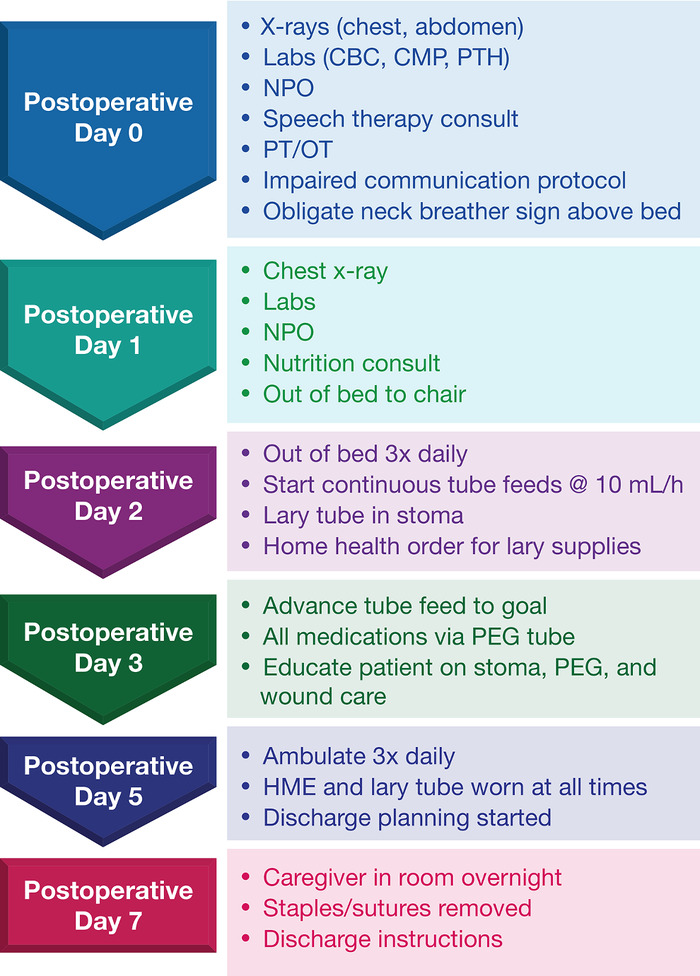
**Flow diagram of laryngectomy clinical pathway.** CBC, complete blood count; CMP, comprehensive metabolic panel; HME, heat moisture exchange; lary tube, laryngectomy tube; NPO, nothing by mouth; PEG, percutaneous gastrostomy; PT/OT, physical therapy/occupational therapy; PTH, parathyroid hormone.

**Table 1.** Detailed Laryngectomy Pathway Orders

Intervention Type	Postoperative Day 0	Postoperative Day 1	Postoperative Day 2	Postoperative Day 3	Postoperative Day 4	Postoperative Day 5	Postoperative Day 6	Postoperative Day 7
Outcomes	Mechanical vent weaned in OR	Out of bed to chair once	Out of bed 3 × Lary tube worn at all times (if applicable)	Out of bed 3 × HME and lary tube worn at all times	Out of bed 3 × Ambulate 3 × HME and lary tube worn at all times	Ambulate 3 × HME and lary tube worn at all times Discharge planning	Ambulate 3 × HME removal and replacement Stoma suctioning Stoma care	Caregiver in room with patient overnight and provides care Patient and family demonstrate that patient can safely go home Patient and family verbalize understanding of discharge/medication instructions
Diagnostics	Chest and abdominal x-rays CBC CMP PTH Prealbumin Ionized calcium	Chest x-ray CBC CMP PTH Ionized calcium				CBC		
Treatments	SLP evaluation and treatment PT/OT evaluation and treatment No ties around neck (if flap) Keep head in neutral position (if flap) Flap checks every hour Tracheostomy tube in stoma Stoma care by RT Strip bulb suction and record Neurovascular checks on donor site	Humidified air via tracheostomy collar Clean incisions with saline and apply bacitracin Inpatient consult to hematology/oncology psychology Continue impaired communication protocol	Place HME and change daily Flap checks every 2 hours All medications per tube Discontinue arterial line	Place HME and change daily All medications per PEG tube		OK for ties No ties around neck sign may be taken down Flap checks every 4 hours Neurovascular checks on donor site every shift Tegaderm off STSG donor site		Start rooming in Staples/sutures removed prior to discharge if patient not previously radiated
	Supplies needed at bedside: suction, Ambu bag, duplicate trach obturator, gauze, suture removal kit RT evaluation Answer all call lights in person Communication board in patient room Sign above bed No neck ties Patient is a neck breather; no oral intubation Start impaired communication protocol No pressors unless cleared by surgeon PEG to gravity							
Medications	Duo nebs q4h Morphine or Dilaudid PRN Unasyn or clindamycin and ciprofloxacin Famotidine Synthroid Ondansetron Metoclopramide PRN Promethazine PRN IVF infusion	Bacitracin ointment PCA if needed Melatonin or zolpidem		Hycet via tube	Stop antibiotics			
Activity	Bed rest Head of bed elevated	Progressive mobility protocol						
Diet/Nutrition	Diet NPO	Inpatient consult to dietitian/nutritionist Daily recorded weight	Start continuous TF at 10 mL/h for 24 hours Decrease IVF to keep constant total intake	Increase TF to target				
Prophylaxis	TED hose SCD Heparin or Lovenox							
Education			Laryngectomy training	Stoma care HME PEG care Wound care	Continue stoma, HME, PEG, and wound care	Twice daily: placing and removing HME, stoma suctioning, stoma care		

CBC, complete blood count; CMP, comprehensive metabolic panel; duo nebs q4h, nebulized ipratropium bromide and albuterol sulfate every 4 hours; HME, heat moisture exchange; IVF, intravenous fluid; lary tube, laryngectomy tube; NPO, nothing by mouth; OR, operating room; PCA, patient-controlled anesthesia; PEG, percutaneous gastrostomy tube; PRN, as needed; PT/OT, physical therapy/occupational therapy; PTH, parathyroid hormone level; RT, respiratory therapist; SCD, sequential compression device; SLP, speech and language pathology; STSG, split thickness skin graft; TED, thromboembolic deterrent; TF, tube feeding.

### Data Sources and Measurements

Patient information was obtained from manual review of the EMRs at the Ochsner Clinic Foundation by 3 investigators (S.B.C, S.M.P., and Z.A.F.) for all patients meeting inclusion criteria. Specific data were extracted for patient demographics (age, sex, and race), medical history, perioperative details, and postoperative care. Specific medical history included prior chemotherapy or radiation therapy and medical comorbidities. The Charlson Comorbidity Index (CCI) and the National Surgical Quality Improvement Program (NSQIP) surgical risk were calculated for each patient.^13,14^ The perioperative data included total operative time and whether or not free tissue transfer was used for reconstruction. Postoperative outcome measures included intensive care unit (ICU) LOS, total hospital LOS, ICU readmissions, 30-day readmission rates, and postoperative complications. Postoperative complications included development of a wound complication (including pharyngocutaneous fistula formation) or hematoma, operative reexploration or intervention, cardiovascular complications, sepsis, and death.

### Outcomes and Analysis

The primary outcome was defined as hospital LOS measured as a continuous variable in days. Secondary outcomes were clinical complications, ICU LOS, ICU readmissions, and 30-day readmissions. To quantify the difference in prevalence between the prepathway and postpathway groups, we report risk ratios (RR) and 95% CIs for categorical outcomes. Effect sizes and 95% CIs were calculated using bias-corrected Hedges *g* statistic for all continuous variables. For binary comparison of hospital LOS, we established 12 days as the expected LOS for total laryngectomy procedures. This expected value is based on an absolute minimum stay of 7 days, with an additional 5 days to account for securing the patient's social work needs. The effect on LOS was compared to use of the pathway, use of free tissue transfer, prior treatment history (chemotherapy or radiation therapy), CCI, and the NSQIP risk score.

All patients had complete datasets and were not excluded from the analysis. SAS statistical software, version 9.4 (SAS Institute Inc) was used to conduct all analyses.

This quality improvement study involving protected patient information was approved by the Ochsner Clinic Foundation Institutional Review Board (IRB #2018.240) prior to data collection. The SQUIRE (Standards for QUality Improvement Reporting Excellence) reporting recommendations for quality improvement studies were used to ensure adequate reporting of findings.^15^

## RESULTS

A total of 63 patients met inclusion criteria for the study, and all had follow-up data for analysis. The majority of the patients were white (69.8%) and male (82.5%), and the median age at time of surgery was 65.0 years (interquartile range [IQR] 59.0-73.0 years).

Twenty-nine (46.0%) patients were in the prepathway cohort, and 34 (54.0%) patients were in the postpathway cohort. Patients in the 2 groups were similar with respect to patient demographics, use of alcohol or tobacco, and prior chemotherapy or radiation therapy. The postpathway cohort had an increased percentage of free tissue transfer procedures compared with the prepathway cohort (52.9% vs 20.7%) (Table 2).

**Table 2.** Baseline Demographics Overall and by Cohort

Variable	All Patients, n=63	Prepathway Cohort, n=29	Postpathway Cohort, n=34	*P* Value
Age, years, median (IQR)	65.0 (59.0-73.0)	66.0 (61.0-74.0)	64.0 (59.0-71.0)	0.265
Sex				0.533
Male	52 (82.5)	23 (79.3)	29 (85.3)	
Female	11 (17.5)	6 (20.7)	5 (14.7)	
Race				0.130
White	44 (69.8)	23 (79.3)	21 (61.8)	
Black	19 (30.2)	6 (20.7)	13 (38.2)	
Smoking history				0.836
Never smoker	3 (4.8)	1 (3.4)	2 (5.9)	
Former smoker	37 (58.7)	18 (62.1)	19 (55.9)	
Current smoker	23 (36.5)	10 (34.5)	13 (38.2)	
Alcohol history^a^				0.067
Never drinker	40 (66.7)	23 (79.3)	17 (54.8)	
Former drinker	6 (10.0)	3 (10.3)	3 (9.7)	
Current drinker	14 (23.3)	3 (10.3)	11 (35.5)	
Prior radiation therapy	31 (49.2)	15 (51.7)	16 (47.1)	0.712
Prior chemotherapy	17 (27.0)	8 (27.6)	9 (26.5)	0.921
Charlson Comorbidity Index,^b^ median (IQR)	8.0 (8.0-10.0)	8.0 (8.0-9.0)	8.5 (7.0-10.0)	0.726
NSQIP surgical risk,^c^ median (IQR)	29.7 (27.6-32.6)	28.8 (26.0-33.4)	30.3 (28.6-32.5)	0.241
Operative variables				
Free tissue transfer	24 (38.1)	6 (20.7)	18 (52.9)	0.018
Operative duration, min, median (IQR)	435.0 (375.0-630.0)	390.0 (330.0-495.0)	495.0 (390.0-670.0)	0.053

^a^Alcohol history was not available for 3 patients in the postpathway group.

^b^The Charleson Comorbidity Index is designed to predict 1-year mortality based on a patient's comorbidities. A higher score indicates a higher risk of death.

^c^The National Surgical Quality Improvement Program (NSQIP) surgical risk calculator estimates the risk of postoperative complications based on preoperative data including patient age, body mass index, and comorbid conditions. A higher score indicates a higher risk of complications.

Note: Data are presented as n (%) unless otherwise noted.

IQR, interquartile range; NSQIP, National Surgical Quality Improvement Program.

Both cohorts had a median hospital LOS of 10 days (*P*=0.647). The median ICU LOS of 3 days for the postpathway cohort was significantly higher than the 2 days for the prepathway cohort, but the rates of ICU readmission were not different between the groups (RR=0.4; 95% CI 0.1-1.5).

Because the data for hospital and ICU LOS were skewed, RR could not be calculated, so the hospital LOS was treated as a binary entity with a cutoff of 12 days as the expected LOS. RR (95% CI) for the comparison of hospital LOS <12 days and >12 days was calculated to be 0.9 (0.5-1.4). Multivariate analysis controlling for morbidity indices—CCI and NSQIP risk—showed no difference in LOS >12 or <12 days.

The 30-day readmission rate was similar between cohorts (RR=1.4; 95% CI 0.9-2.2). Overall, we found a reduction in the rate of total complications among the postpathway cohort compared with the prepathway group (RR=0.5; 95% CI 0.3-1.0), although the differences in individual complications were similar. Table 3 presents outcomes and complications data.

**Table 3.** Postoperative Outcomes by Cohort

Postoperative Outcome	Prepathway Cohort, n=29	Postpathway Cohort, n=34	Risk Ratio (95% CI)	*P* Value
Hospital LOS, days, median (IQR)	10 (9-16)	10 (8-19)		0.647
ICU LOS, days, median (IQR)	2 (1-3)	3 (2-4)		<0.001
ICU readmission	6 (20.7)	2 (5.9)	0.4 (0.1-1.5)	0.079
30-day readmission	3 (10.3)	7 (20.6)	1.4 (0.9-2.2)	0.488
Postoperative complications				
Wound complication	9 (31.0)	4 (11.8)	0.5 (0.2-1.2)	0.060
Operating room reexploration	5 (17.2)	5 (14.7)	0.9 (0.3-2.7)	0.784
Hematoma	1 (3.4)	1 (2.9)	0.9 (0.2-3.8)	0.909
Cardiovascular complication	5 (17.2)	1 (2.9)	0.2 (0.0-1.4)	0.054
Sepsis	2 (6.9)	0 (0.0)	0.5 (0.1-2.5)	0.120
Death	1 (3.4)	0 (0.0)	0.4 (0.0-4.5)	0.275
Overall postoperative complication rate^a^	13 (44.8)	7 (20.6)	0.5 (0.3-1.0)	0.030

^a^Defined by the occurrence of a patient having >0 complications.

Note: Data are presented as n (%) unless otherwise noted.

ICU, intensive care unit; LOS, length of stay.

## DISCUSSION

Our study demonstrates a decreased overall complication rate with the implementation of a postoperative clinical care pathway in a cohort of patients undergoing total laryngectomy. However, we found no difference in hospital LOS or in ICU or 30-day readmission rates.

As rates of laryngectomies performed across the United States have decreased, performance of these procedures has become concentrated at high-volume academic centers.^2,6,16^ These specialized institutions must be equipped to properly care for this unique group of patients through education of the patient, family, and staff and by implementing measures that streamline care while safeguarding against potential complications.

Verma and Mahboubi examined trends in total laryngectomies performed in the state of California between 1996 and 2010, and similar to national trends, found an overall decrease in total laryngectomy procedures performed during this period, along with an increasing proportion performed at tertiary referral centers.^16^ Accompanying this trend, the authors found an increase in the overall complication rate. The authors hypothesized that the higher complication rate could be explained by the greater complexity of total laryngectomies with changing treatment protocols and the increasing number of salvage (postradiated) surgeries. Contrary to the findings of the Verma and Mahboubi study, our data demonstrate a decrease in total complication rates associated with total laryngectomies between January 2016 and December 2018. Our finding perhaps indicates a protective effect of the laryngectomy pathway, safeguarding against potential risks that patients undergoing total laryngectomy face postoperatively.

Clinical pathways have been proven to reduce resource utilization without jeopardizing safety.^8-12^ In 1999, Hanna et al examined how a clinical pathway impacted cost and quality of care for postoperative laryngectomy patients at their institution.^11^ The study showed a 14.4% reduction in hospital cost associated with pathway implementation, as well as a decrease in readmission rate. Our study, while similar in design to that of Hanna et al, differs in that our postintervention population is more than double the size and includes patients reconstructed with free tissue transfer techniques. While we did not examine cost, we found no significant difference between the groups in terms of readmission rate. Our data provide a contemporary example of the use of a clinical pathway for patients undergoing laryngectomy.

Our results echo the positive findings of other studies examining the effects of clinical pathways on patient outcomes.^8-10,12^ We found a decrease in the total complication rate in the postpathway cohort. Thus, the prepathway group was at increased risk of having a complication overall even though individual complication rates did not differ significantly between the 2 cohorts. The pathway did not appear to have an effect on overall hospital LOS, even when controlling for comorbidities using the NSQIP and CCI.

Use of free tissue transfer to reconstruct laryngectomy defects has increased at our institution since December 2015, coinciding with the implementation of the laryngectomy pathway. Free flaps, chiefly from the anterolateral thigh, are used for reconstruction in patients with a history of radiation and for bulky primary disease. Free flaps have been proven to reduce fistula rates, feeding tube dependence, and risk of remote esophageal stricture in patients undergoing total laryngectomy who have failed organ preservation treatment.^17^ Patients who undergo free tissue transfer for defects of the head and neck typically stay in the ICU for an average of 2.4 days,^18^ and those who have free flap reconstruction after total laryngectomy have a longer LOS than those not requiring free tissue transfer.^19^ A significantly higher percentage of free flaps was used for reconstruction in our postpathway group. This bias in our study could help to explain the increased ICU LOS in the postpathway group and could have mitigated the decrease in overall hospital LOS. In other words, we could have expected a significant increase in LOS given the use of more free flaps; however, perhaps the pathway helped prevent such an increase.

This study has several limitations. Primarily, the retrospective design predisposes our results to reporting bias based on the accuracy of diagnosis classification in the EMR. Reporting bias may lead to inaccurate representation of complications, as they may have been inappropriately recorded. The outcome of LOS is less likely to be affected by reporting bias, although multiple confounding variables may alter the LOS, including patient comorbidities, prior radiation therapy or chemotherapy, and nutrition status. To control for confounding variables, we performed multivariate analyses based on patient comorbidities and surgical risk and found no difference between groups in reference to overall hospital LOS. For the binary comparison of hospital LOS between the groups, we chose the number 12 arbitrarily, although we felt that 12 days was a reasonable expected LOS given an absolute minimum of 7 days and 5 days to accommodate social factors. Further, selection bias may arise as the 2 cohorts are from different time periods. Surgeon expertise likely improves over time, which would affect the total complication rate in the postpathway group. Further follow-up of postpathway cohorts is necessary to confirm the trends found in this study.

## CONCLUSION

The implementation of a postoperative order set pathway for patients undergoing total laryngectomy was associated with a decrease in the overall complication rate at our institution. In an era of declining use of total laryngectomy and higher concentration of total laryngectomies performed at high-volume academic centers, measures that standardize care and improve outcomes should be implemented when possible.

## Notes

*This article meets the Accreditation Council for Graduate Medical Education and the American Board of Medical Specialties Maintenance of Certification competencies for Patient Care, Medical Knowledge, Systems-Based Practice, and Practice-Based Learning and Improvement.*
